# Remote Ischemic Conditioning With Exercise (RICE)—Rehabilitative Strategy in Patients With Acute Ischemic Stroke: Rationale, Design, and Protocol for a Randomized Controlled Study

**DOI:** 10.3389/fneur.2021.654669

**Published:** 2021-05-03

**Authors:** Zhenzhen Han, Wenbo Zhao, Hangil Lee, Melissa Wills, Yanna Tong, Zhe Cheng, Qingqing Dai, Xiaohua Li, Qingzhu Wang, Xiaokun Geng, Xunming Ji, Yuchuan Ding

**Affiliations:** ^1^Department of Neurology, Beijing Luhe Hospital, Capital Medical University, Beijing, China; ^2^Xuanwu Hospital, Capital Medical University, Beijing, China; ^3^School of Medicine, Wayne State University, Detroit, MI, United States

**Keywords:** acute ischemic stroke (AIS), exercise rehabilitation, remote ischemic conditioning (RIC), neuroprotection, intravenous thrombolysis

## Abstract

**Objective:** Exercise rehabilitation is an effective therapy in reducing the disability rate after stroke and should be carried out as early as possible. However, very early rehabilitation exercise exacerbates brain injury and is difficult to conduct in stroke patients due to their weakened and potentially disabled state. It is valuable to explore additional early rehabilitation strategies. Remote Ischemic Conditioning (RIC) is a novel therapy designed to protect vital organs from severe lethal ischemic injury by transient sublethal blood flow to non-vital organs, including the distal limbs, in order to induce endogenous protection. RIC has previously been conducted post-stroke for neuroprotection. However, whether combined early RIC and exercise (RICE) therapy enhances stroke rehabilitation remains to be determined.

**Methods:** This is a single-center, double-blinded, randomized controlled trial that will enroll acute ischemic stroke patients within 24 h of symptom onset or symptom exacerbation. All enrolled patients will be randomly assigned to either the RICE group (exercise with RIC) or the control group (exercise with sham RIC) at a ratio of 1:1, with 20 patients in each group. Both groups will receive RIC or sham RIC within 24 h after stroke onset or symptom exacerbation, once a day, for 14 days. All patients will begin exercise training on the fourth day, twice a day, for 11 days. Their neurological function [Modified Rankin Scale (mRS) score, National Institutes of Health Stroke Scale (NIHSS) score, Barthel Index, and walking ability], infarct volume (nuclear magnetic resonance, MRI), and adverse events will be evaluated at different time points in their post-stroke care.

**Results:** The primary outcome is safety, measured by the incidence of any serious RICE-related adverse events and decreased adverse events during hospitalization. The secondary outcome is a favorable prognosis within 90 days (mRS score < 2), determined by improvements in the mRS score, NIHSS score, Barthel Index, walking ability after 90 days, and infarct volume after 12 ± 2 days.

**Conclusion:** This study is a prospective randomized controlled trial to determine the rehabilitative effect of early RIC followed by exercise on patients with acute ischemic stroke.

**Trial Registration:** www.chictr.org.cn, identifier: ChiCTR2000041042

## Introduction

Stroke is a leading cause of death and disability worldwide (1, 2), with an annual mortality rate of approximately 5.5 million (3). Through the application of evidence-based control measures, the burden of stroke death has declined in many developed countries—in the Western world, death from stroke declined by 30–50% from 1975 to 2005 (4). However, despite an increase in the rate of effective life-saving interventions, such as vascular recanalization by intravenous thrombolysis and intravascular therapy, over 50% of the survivors are left with new disabilities (5). Therefore, stroke remains as a disease of enormous public health importance, and the need for an effective stroke rehabilitation beyond mortality reduction is growing as an essential part of the continuum of stroke care. Acute ischemic stroke, in which an embolic or thrombotic event occludes an artery supplying the brain, accounts for 80% of all strokes (4) and thus represents an unmatched source of devastating disability (6).

Stroke protocols are carried out within hours of the cerebrovascular accident in order to maximize penumbral reperfusion and tissue recovery (7–9). Analogously, rehabilitation techniques, which can also enhance reperfusion and are an indispensable component of promoting the functional recovery of stroke patients, should be carried out as early as possible. Numerous clinical studies have demonstrated that exercise enhances motor function after a cerebrovascular accident (10). Therefore, national guidelines for improving stroke outcomes recommend early exercise rehabilitation (11). However, A Very Early Rehabilitation Trial (AVERT) demonstrated that very early mobilization (<24 h) after stroke actually exacerbated brain injury and reduced rates of favorable prognoses measured at 3 months (12, 13). Similar results were also found in our clinical study (14). Furthermore, patients at the early stages of recovery from stroke had unstable body conditions and poor endurance and found it difficult to adapt to exercise training, which limited the promotion of early exercise rehabilitation after stroke (15). Moreover, since patients differ in the nature of their cerebrovascular accidents and resulting disabilities; it is difficult to recommend and implement standardized rehabilitation protocols that are both safe and effective for all stroke patients. These findings point to a gap in the continuum of stroke care at its early recovery stages: theoretically, early exercise intervention is beneficial to stroke victims, but the practicality of exercise rehabilitation limits its clinical applications. This gap calls for exploration of different novel, simple, and feasible rehabilitation models to be implemented in the early stages of recovery—when exercise poses greater harm than good.

Remote ischemic conditioning (RIC) is a novel therapy that was initially developed in the realm of cardioprotection after myocardial infarction, where it was shown to reduce infarct size, minimize ischemia/reperfusion injury, and prevent the onset of heart failure (16). More recently, it has emerged for victims of cerebrovascular accidents due to its non-invasive, easy-to-administer, and low-cost nature (17). RIC involves the induction of transient sublethal ischemia *via* controlled blood flow restriction to the distal limbs, which induces endogenous protective effects against severe lethal ischemic injury to vital organs of the body, such as the heart, brain, and kidney (18). RIC has been shown to function by similar biochemical mechanisms as physical exercise, such as through the promotion of neurogenesis and angiogenesis, which are essential for post-stroke rehabilitation (19, 20).

Studies have demonstrated RIC to be an effective prophylactic therapy for acute ischemic stroke in patients with symptomatic intracranial arterial stenosis (SIAS) (21) and during carotid artery stent placements (22). In post-stroke therapy, RIC has been shown to enhance cognitive function, particularly in the domains linked to visuospatial, executive functioning, and attention (23). Furthermore, long-term application of RIC appears to have the same benefits to the human body as exercise (24) and has been shown to improve walking speed and to reduce neuromuscular fatigue in chronic stroke survivors (25). These studies and others suggest that the use of RIC in stroke patients enables a similar spectrum of benefits as exercise therapy without many of its associated drawbacks. In contrast to physical activity, RIC is administered to the patient passively and depends less on his or her motivation, level of physical activity, and degree of post-stroke disability, especially at the early stage. Indeed, RIC is performed through the basic application of a blood pressure cuff to the arm for sessions measured in minutes, and is thus feasible for patients irrespective of their clinical picture (26). However, research on RIC is in its infancy, and it is still unclear whether it can be used as a unique component, or eventually as the sole therapy, of early rehabilitation to improve neurological function after stroke.

In this study, we will evaluate the safety and feasibility of RIC with exercise (RICE) as a novel rehabilitation strategy in patients with acute ischemic stroke. RIC, a simple procedure, will be implemented as early as possible to initiate rehabilitation closer to the onset of the ischemic event and into the time frame in which early exercise rehabilitation was shown to exacerbate brain injury. It will be combined with exercise, implemented at the time at which it has been shown to promote functional recovery and improve long-term functional prognosis. Clinical improvement with very early RIC would highlight RIC as an effective rehabilitation method that could bridge the gap in the continuum of very early post-stroke care. Marked improvement in the dual therapy group could also point to an additive or synergistic effect of exercise and RIC therapy, namely, the RICE.

## Methods

### Study Design

This is a single-center, double-blinded, randomized controlled trial that will enroll acute ischemic stroke patients within 24 h of the ischemic event or symptom exacerbation. All participants will be informed about the clinical study and the requirement to give informed consent. The study protocol and informed consent were approved by the regional ethics committee and have been registered with the Clinicaltrials.gov (ChiCTR2000041042).

After receiving informed consent, all enrolled patients will be randomly assigned to either the intervention group (RIC with exercise rehabilitation) or the control group (sham RIC with exercise rehabilitation) at a ratio of 1:1, with 20 patients in each group. Within 24 h of the ischemic event or symptom exacerbation, the groups will start either RIC or sham RIC in 45-min sessions, once a day, for 14 days. All patients will begin exercise rehabilitation training 4 days after the ischemic stroke event in 30-min sessions, twice a day, for 11 days. Brain imaging with MRI will be performed on the day of the stroke for a baseline and again at 12 days. Evidence of stroke severity and disability will be evaluated using the National Institutes of Health Stroke Scale (NIHSS) score and the Barthel Index, which will be administered by trained investigators blinded to the treatment assignment on the day of the stroke for a baseline and at 1, 3, 7, and 90 days after enrollment. Furthermore, the Modified Rankin Scale (mRS) will be administered, and walking ability will be assessed at 90 days.

### Patient Population

Participants will be recruited from the hospital wards. The inclusion criteria for recruitment are as follows: (1) age: 18–80 years old; (2) patients with confirmed acute ischemic stroke (mRS ≤ 2 and NIHSS score: 6–16), including those who received intravenous thrombolysis or mechanical embolectomy; (3) randomized grouping ≤24 h of stroke onset or symptom exacerbation; and (4) written informed consent provided by the participant or legally authorized representative. The exclusion criteria for recruitment include the following: (1) contraindications for ischemic conditioning (e.g., severe soft tissue injury, fracture, and peripheral vascular disease in both upper limbs); (2) unstable vital signs (e.g., systolic blood pressure <120 or >220 mmHg, heart rate <40 beats/min or >100 beats/min, percutaneous oxygen saturation ≤92%, body temperature ≥38.5°C); (3) lower limb fracture(s) or other factors that would prevent exercise training completion; (4) history of poor compliance; (5) life expectancy ≤1 year; (6) severe hepatic, pulmonary, and/or renal dysfunction; (7) coagulation dysfunction or active bleeding; (8) combined acute coronary syndrome or severe arrhythmia; (9) pregnant or lactating patients; and/or (10) participation in another clinical trial currently or within 30 days before study inclusion.

### Randomization

All enrolled patients will be randomly assigned to either the intervention group or the control group at a ratio of 1:1. Randomized sequence column orders will be made according to a predefined table generated by a computer program. The random sequence will be hidden in an enclosed opaque envelope. After the baseline patient data are collected by a specialized member of the research personnel, subjects will be randomly assigned to either the intervention group or the control group.

### Interventions

RIC will be performed by placing an electronic tourniquet around both arms within 24 h of stroke symptom onset or symptom exacerbation. Participants in the intervention group will undergo five cycles of cuff inflation to 200 mmHg for 5 min, followed by deflation for 5 min. This will be repeated once daily for the subsequent 14 days. Patients in the sham RIC group will receive the same procedure as the treatment group, but the maximal inflation pressure will be set to only 60 mmHg.

**Figure 1:**
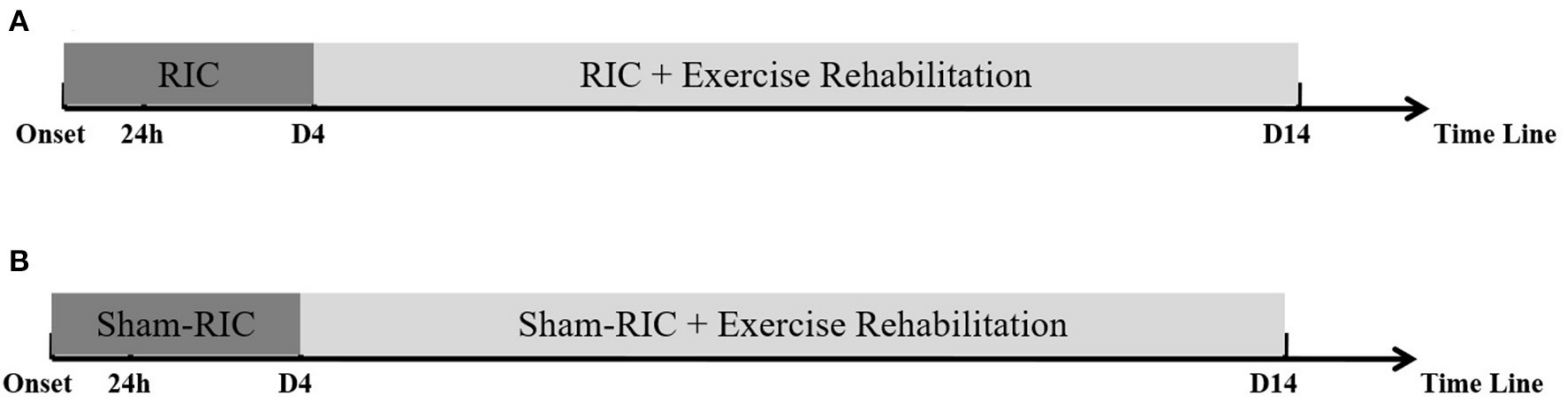
Procedure timeline for experimental protocol. **(A)** RICE group (RIC with exercise); **(B)** Control group (Sham-RIC with exercise).

All patients will receive daily out-of-bed exercise training twice a day for 30 min, starting from 4 days after symptom onset, for 11 days. Out-of-bed mobilization, as described previously by us (14), will include sitting, standing, and walking, which will be performed with or without assistance. While no special equipment will be used, mobilization will permit the use of standing bed and wheelchair when necessary. All mobilization protocols will be adjusted to each individual patient's tolerance, needs, and abilities and will be delivered by professional therapists or nurses. The frequency, dose, and content of mobilization will vary according to the individual patient's physical ability and will be recorded in detail by therapists or nurses. The dose of exercise will be monitored by a specially assigned staff to ensure good compliance for this study. Physicians will be asked to evaluate patients with deteriorating conditions during the exercise and to postpone mobilization when necessary (14). Patients in both groups will receive standard stroke treatment according to the guidelines, including thrombolysis, anti-platelet aggregation, and lipid reduction (Figure 1).

Early RIC combined with follow-up exercise training is a novel early rehabilitation model for acute stroke patients; therefore, we set the duration of RIC and exercise (RICE) rehabilitation to be 14 days according to the protocol of “A Very Early Rehabilitation Trial” (AVERT) (13), the well-known and evidence-based clinical study on early rehabilitation of stroke, and our previous study (14).

### Outcomes

#### Primary Outcome

The primary outcome is safety, including any serious RIC-related and exercise-related adverse events. Other safety outcomes include clinical deterioration, recurrence of stroke, fall, angina, myocardial infarction, deep venous thrombosis, pulmonary embolism, pressure sore, chest infection, urinary tract infection, and depression during hospitalization. All adverse events will be determined independently by specially trained members of the same research group who will be blinded to the randomization. Source data will be reviewed if necessary.

#### Secondary Outcomes

There will be two classes of secondary outcomes. (1) Clinical efficacy as determined by the mRS (mRS 0–2 was defined as a favorable prognosis and mRS 3–6 points as a poor prognosis), NIHSS score, Barthel scale, and the proportion of patients achieving independent walking after 90 days (Holden functional classification of walking). (2) Brain infarct volume as determined by MRI diffusion-weighted imaging technique, with the lesion profile plotted at each individual level by an image tool on the workstation to calculate the area. The levels will be multiplied by the thickness of each level and summed to calculate the infarct volume. The calculations will be performed by personnel blinded to clinical data and randomization at baseline and at day 12 ± 2.

### Sample Size Estimation

This is a phase 1 safety and feasibility trial. There are no data available for reference because no clinical study of RIC and exercise therapy in patients with confirmed acute ischemic stroke (mRS ≤ 2, NIHSS score 6–16) has yet been completed. Hertzog (27) has suggested that 10–20 patients in each group are sufficient to assess the feasibility of a pilot study, while Dobkin (28) has shown that 15 patients in each group are usually enough to decide whether a larger multicenter trial should be conducted. A similar number of samples was selected in two other related protocols (29) (Lv et al., Frontiers in Neurology in press ). We therefore set our recruitment goal to 20 patients per group in the present study. The results of this study will be used to determine the safety and feasibility of RICE and be used to estimate the sample size and perform power calculations necessary to plan the phase 2 trials.

### Statistical Analysis

All statistical analyses will be performed using the statistical software SAS (version 9.1.3; SAS Institute, Cary, NC). Outcome event analyses will be based on the intention-to-treat (ITT) principle, including all randomly enrolled subjects. Subjects' baseline categorical variables will be recorded in percentages (%), and continuous variables will be recorded as means and standard deviations or medians and interquartile ranges. The comparison of baseline parameters of the study subjects will be performed with the Pearson chi-square test and either the *t*-test or the Wilcoxon rank sum test for the categorical and continuous variables, respectively.

The distribution difference of the 90-day mRS scores between the two groups will be evaluated using the common ratio. The public odds ratio of the mRS scores on the 90th day will be evaluated by the sequential logistic regression model. The ratios between the mRS categories (0–2 and 3–6) and adverse events will be estimated with the improved Poisson regression. Infarct volume and β coefficient of the NIHSS score will be estimated by multiple linear regressions. Efficacy assessment will be adjusted based on age, gender, NIHSS baseline score, baseline condition, stroke subtype, intravenous alteplase treatment, and mechanical thrombectomy. The homogeneous dominance ratio of each subgroup will be analyzed *via* the Breslow–Day test to analyze the functional independence of the subgroup for 90 days. The significance level will be set at 0.05 for all tests.

## Discussion

Exercise rehabilitation has been confirmed to be one of the most effective approaches to improving prognosis and preventing lasting complications after stroke (30). It is an indispensable component in the organizational management of cerebrovascular diseases (31). The latest domestic and international guidelines for stroke rehabilitation recommend early rehabilitation for patients with acute stroke (11). However, the optimal model of early rehabilitation is still controversial. Recent studies have confirmed that very early exercise rehabilitation exacerbates brain injury (12). The promotion of very early exercise rehabilitation is also limited in actual clinical practice for many reasons, such as unstable vital signs, low cardiorespiratory fitness, and poor muscle strength and muscle power in stroke patients (15).

Ischemic conditioning is an approach that provides neuroprotection (32) and has been observed to be efficacious in promoting rehabilitation for patients with global cerebral ischemia (33). The use of ischemic conditioning has been clinically demonstrated through hypobaric and normobaric hypoxia (34, 35). RIC has also been used widely in other contexts (36), including reduction of myocardial injury after ischemia in large animal models and human trials (37). In studies of cerebral pathologies, RIC has improved cognition in patients with subcortical ischemic vascular dementia (38) and conferred neuroprotective effects after acute ischemic stroke (39). It offered neuroprotection through enhanced cerebral perfusion, cerebral collateral formation, and tolerance of cerebral ischemia (40, 41). Furthermore, studies have shown that long-term RIC training can reduce nerve injury (42), promote nerve remodeling and angiogenesis (25), and promote the motor function of paralyzed limbs (43, 44). Chronic and repetitive RIC has been applied to clinical trials and is expected to exert its protective role against cerebral ischemia and repeated stroke in a long-term fashion (40). It has also been demonstrated to improve performance in sports medicine, akin to the physiological improvement that is appreciated in routine exercise training (24). Some underlying mechanisms of RIC and exercise have been evidenced to overlap—both therapies demonstrate increased expression of heat shock proteins, enhanced involvement of the nitric oxide (NO) pathway, modification of ATP-sensitive potassium (K_ATP_) channels' functionality, enhanced antioxidant capacity, induction of autophagy, involvement of the opioid system, and regulation of the immune and inflammatory system (24). Together, these processes enhance neurogenesis, angiogenesis, and defense against oxidative stress in the brain after an ischemic event.

RIC and exercise have similar temporal windows in which they induce benefits in the rehabilitative stage of stroke recovery. They also have similar effects and mechanisms in performance improvement. These overlapping biochemical and clinical characteristics appoint RIC as a suitable candidate to fill the “gap” that is seen in very early rehabilitation, of which physical exercise is currently the sole therapy. Using RIC for the very early time frame would avoid the deleterious impact of exercise while potentially reaping RIC's unique benefits including ischemia and hypoxia tolerance. In other words, the combination of novel early RIC training followed by exercise rehabilitation, which is known to be effective, could form a new type of early stroke rehabilitation model. The model would enable the mechanisms of exercise therapy known to be beneficial to stroke patients, such as increased neurogenesis and angiogenesis in the brain, to be initiated earlier than is normally feasible. The subsequent application of traditional exercise therapy would reinforce these processes and lead to greater clinical progress and better prognosis after several months. This novel rehabilitation strategy will be applied for the investigation of functional recovery promotion, disability reduction, and prognosis improvement in patients with ischemic stroke.

The NIHSS score has been demonstrated to be a good prognostic indicator of stroke outcome (45). NIHSS scores of ≤6 indicate a high likelihood of good prognosis; scores of 7–10 and 11–15 indicate good prognosis rates of 46 and 23%, respectively (45); and scores of ≥16 suggests a poor prognosis and a high possibility of death or severe disability (46). Patients with disabilities after stroke mostly have NIHSS scores >6, which was set as the lower bound for our inclusion criteria in this study. Moreover, it has been reported by previous studies that it is difficult for patients with severe stroke-induced disability (NIHSS score ≥16) to perform early exercise rehabilitation due to various reasons, such as severe symptoms, unstable conditions, and intolerance of early out-of-bed rehabilitation treatment (15). Therefore, patients with NIHSS scores >16 will be excluded from this study. To summarize, moderate acute ischemic stroke patients with NIHSS scores of 6–16 will be included in this study, as these patients will likely be able to participate in the given tasks for rehabilitation and subsequently have a favorable prognosis.

RIC is a non-invasive, feasible, and promising rehabilitative method for patients recovering from ischemic stroke. It has been demonstrated that post-stroke RIC training can be carried out safely within 6–24 h of acute ischemic stroke attack and induce a significant neuroprotective and neurorehabilitative effect (39). Hence, RIC training will be initiated within 24 h after stroke onset in this study.

Very early exercise rehabilitation with physical exercise after stroke may aggravate brain injury and reduce the rate of good prognosis at 3 months post-cerebrovascular event (47). Previous expert consensus also recommends that exercise rehabilitation treatment be conducted within 48–72 h after stroke, after which patients have regained stable vital signs and no longer have acutely deteriorating neurological symptoms (12). Therefore, in this study, out-of-bed exercise rehabilitation will be carried out 72 h after stroke attack, that is, on the fourth day of stroke. Early RIC combined with follow-up exercise training is a novel early rehabilitation model for acute stroke patients; therefore, we set the duration of RIC and exercise rehabilitation to be 14 days according to the protocol of “A Very Early Rehabilitation Trial” (AVERT) (48), the most well-known and evidence-based clinical study on early rehabilitation of stroke.

In this way, our study will employ two treatments that are known to be effective in stroke rehabilitation at their respective temporal windows that are known to maximize benefits and reduce adverse events. This temporal optimization will enable stroke patients to benefit from the individual advantage of RIC and exercise rehabilitation and perhaps from a temporal and synergistic role of the two therapies.

There are some limitations to this study. First, as previous research is lacking, the sample size was calculated based on previous relevant literature (27, 28). Additionally, the patient population is highly targeted in this study: we have limited the inclusion criteria to patients with moderate acute cerebral infarction and moderate-to-high disability rate (NIHSS scores 6–16). Therefore, the results may not be accurately generalizable to all patients with acute cerebral infarction. Future directions should seek to apply this modem of post-stroke therapy to patients with infarcts varying in size and severity as well as diverse degrees of post-stroke disability as determined by the NIHSS score.

## Perspective and Prospective

The aim of this study is to clarify the safety and efficacy of early RIC combined with follow-up exercise training as rehabilitation for patients with moderate acute cerebral infarction. The experimental results will reflect a new strategy for stroke rehabilitation that can improve the clinical prognosis of patients with acute cerebral infarction.

## Ethics Statement

The studies involving human participants were reviewed and approved by Medical Ethical Committee of Beijing Luhe Hospital, Capital Medical University. The patients/participants provided their written informed consent to participate in this study.

## Author Contributions

ZH, YT, WZ, HL, MW, and XL prepared the manuscript. ZH, ZC, QD, QW, XJ, YD, and XG designed the study and revised the manuscript. All authors contributed to the article and approved the submitted version.

## Conflict of Interest

The authors declare that the research was conducted in the absence of any commercial or financial relationships that could be construed as a potential conflict of interest.
